# Supplementary material for: Biochemical Evolution of a Potent Target of Mosquito Larvicide, 3-Hydroxykynurenine Transaminase
Source: Molecules. 2022 Aug 2;27(15):4929. doi: 10.3390/molecules27154929 (PMC9369995; doi:10.3390/molecules27154929)
Supplement: Supplementary file 1 [file molecules-27-04929-s001.zip › molecules-1822245-supplementary.pdf]

Supplementary Materials

Table S1. AGT homologous in mosquitoes. Bold font represents putative HKT. The domain matched in the Pfam and Cd-Search database is displayed as Pfam id and Cd-search accession number. PF002666 is Pfam accession number for aminotransferase class-V protein domain. Cd06451 is CD-search accession number for alanine-glyoxylate aminotransferase family.

Organism	Assembly	Sequence ID	Lenth(aa)	Pfam/CD-search domain hit
Aedes aegypti	GCA_002204515.1	XP_001656924.2	400	PF00266/cd06451
Aedes albopictus	GCA_006496715.1	XP_029730871.1	400	PF00266/cd06451
Culex quinquefasciatus	GCA_015732765.1	XP_038113215.1	399	PF00266/cd06451
Anopheles gambiae	GCA_000005575.1	XP_309676.3	396	PF00266/cd06451
Anopheles minimus	GCA_000349025.1	AMIN000902	417	PF00266/cd06451
Anopheles funestus	GCA_003951495.1	AFUN002379	397	PF00266/cd06451
Anopheles stephensi	GCA_013141755.1	XP_035895729.1	397	PF00266/cd06451
Anopheles coluzzii	GCA_016920705.1	XP_040226202.1	396	PF00266/cd06451
Anopheles quadriannulatus	GCA_000349065.1	AQUA001720	397	PF00266/cd06451
Anopheles arabiensis	GCA_016920715.1	XP_040155497.1	397	PF00266/cd06451
Anopheles melas	GCA_000473525.2	AMEC014977	397	PF00266/cd06451
Anopheles merus	GCA_017562075.2	XP_041763925.1	396	PF00266/cd06451
Anopheles christyi	GCA_000349165.1	ACHR002664	384	PF00266/cd06451
Anopheles epiroticus	GCA_000349105.1	AEPI003369	398	PF00266/cd06451
Anopheles farauti	GCA_000473445.2	AFAF001628	364	PF00266/cd06451
Anopheles dirus	GCA_000349145.1	ADIR008341	396	PF00266/cd06451
Anopheles albimanus	GCA_013758885.1	XP_035779445.1	397	PF00266/cd06451
Anopheles darlingi	GCA_000211455.3	ETN58525.1	397	PF00266/cd06451
Anopheles sinensis	GCA_000441895.2	ASIS017663	397	PF00266/cd06451
Anopheles atroparvus	GCA_914969975.1	AATE003211	396	PF00266/cd06451
Anopheles culicifacies	GCA_000473375.1	ACUA009156	396	PF00266/cd06451
Aedes aegypti	**GCA_002204515.1**	**XP_001660875.1**	**393**	**PF00266/cd06451**
Aedes albopictus	**GCA_006496715.1**	**XP_029723387.1**	**393**	**PF00266/cd06451**
Culex quinquefasciatus	**GCA_015732765.1**	**XP_001862936.2**	**393**	**PF00266/cd06451**
Anopheles gambiae	**GCA_000005575.1**	**XP_311559.3**	**396**	**PF00266/cd06451**
Anopheles albimanus	**GCA_013758885.1**	**XP_040168896.1**	**386**	**PF00266/cd06451**
Anopheles darlingi	**GCA_000211455.3**	**ETN65727.1**	**406**	**PF00266/cd06451**
Anopheles merus	**GCA_017562075.2**	**XP_041783722.1**	**396**	**PF00266/cd06451**
Anopheles melas	**GCA_000473525.2**	**AMEC000336**	**216**	**PF00266/cl18945**
Anopheles coluzzii	**GCA_016920705.1**	**XP_040235713.1**	**396**	**PF00266/cd06451**
Anopheles arabiensis	**GCA_016920715.1**	**XP_040168896.1**	**396**	**PF00266/cd06451**
Anopheles quadriannulatus	**GCA_000349065.1**	**AQUA006703**	**542**	**PF00266/cd06451**
Anopheles christyi	**GCA_000349165.1**	**ACHR006122**	**405**	**PF00266/cd06451**
Anopheles epiroticus	**GCA_000349105.1**	**AEPI000688**	**396**	**PF00266/cd06451**
Anopheles funestus	**GCA_003951495.1**	**AFUN002641**	**396**	**PF00266/cd06451**
Anopheles minimus	**GCA_000349025.1**	**AMIN006582**	**396**	**PF00266/cd06451**
Anopheles stephensi	**GCA_013141755.1**	**XP_035892335.1**	**396**	**PF00266/cd06451**

Anopheles farauti	GCA_000473445.2	AFAF004225	406	PF00266/cd06451
Anopheles dirus	GCA_000349145.1	ADIR011618	396	PF00266/cd06451

Table S2. Quantitative structure comparisons using TM-align and Dali server. The structure of AaeHKT is used as a query for pairwise comparison. TM-scores of 0.0-0.30 indicate random structural similarity; TM-scores of 0.5-1.00 indicate that the two structures adopt generally the same fold (1.00 represents a perfect match). Dali Z-scores of < 2 indicate spurious similarity.

	AaeHKT	AgamHKT	AaeAGT	Human AGT	*Mus musculus AGT*	*Nostoc sp.*AGT
Dali Z-score	100	68	62.9	58.3	57.5	52.3
TM-align score	1	0.99393	0.9808	0.98081	0.98237	0.93589

Table S3. Templates searched by I-TASSER. Identity 1 is the percentage sequence identity of the templates in the threading aligned region with the query sequence. Identity 2 is the percentage sequence identity of the whole template chains with query sequence. Coverage represents the coverage of the threading alignment and is equal to the number of aligned residues divided by the length of query protein. Norm. Z-score is the normalized Z-score of the threading alignments. Alignment with a Normalized Z-score >1 mean a good alignment and vice versa [51,59,60].

PDB Hit	Identity 1	Identity 2	Coverage	Norm. Z-score
2CH1A	0.68	0.63	0.93	5.21
7NS7A	0.53	0.44	0.82	2.83
2DR1	0.26	0.28	0.88	1.16
3ISLA	0.36	0.34	0.93	3.72
3KGWA	0.52	0.48	0.93	8.85
3KGXB	0.52	0.49	0.94	3.42

	AaeHKT	AaeAGT
Mosquito HKTs	95	51.43
Coleoptera	55.56	59.17
Mosquito AGTs	51.43	96.18
Blattodea	52.14	58.79
Hymenoptera	51.86	56.17
Vertebrata	50.66	53.16
Arthropoda	50.53	56.73
Thysanoptera	47.49	54.11
Non-Metazoa	45.63	52.88
Bacteria	45.23	48.37
Lepidoptera	42.38	43.02
Archaea	39.67	40.27
Fungi	36.17	31.45
Plants	32.03	32.76

Figure S1. Heatmap of similarity indices between AaeHKT and other mosquito HKTs/other organisms AGTs, and between AaeAGT and other organisms AGTs represented as different taxa listed in the left column. The numbers in the boxes represent the similarity value of the highest similarity hits.

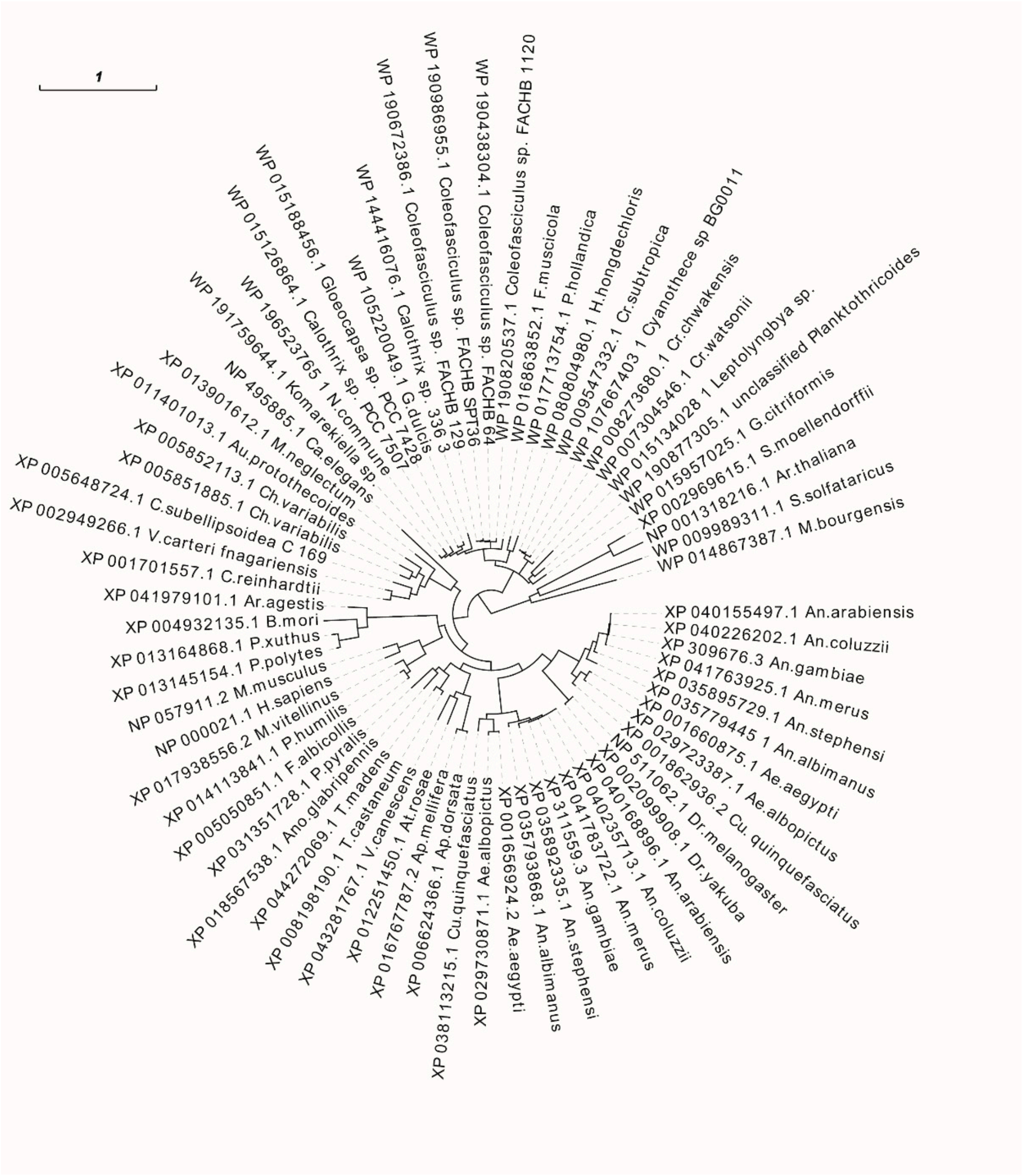

Figure S2. Evolutionary relationships among amino acid sequences of AGT. The evolutionary history was inferred by Bayesian analysis. The tree was rooted by midpoint.

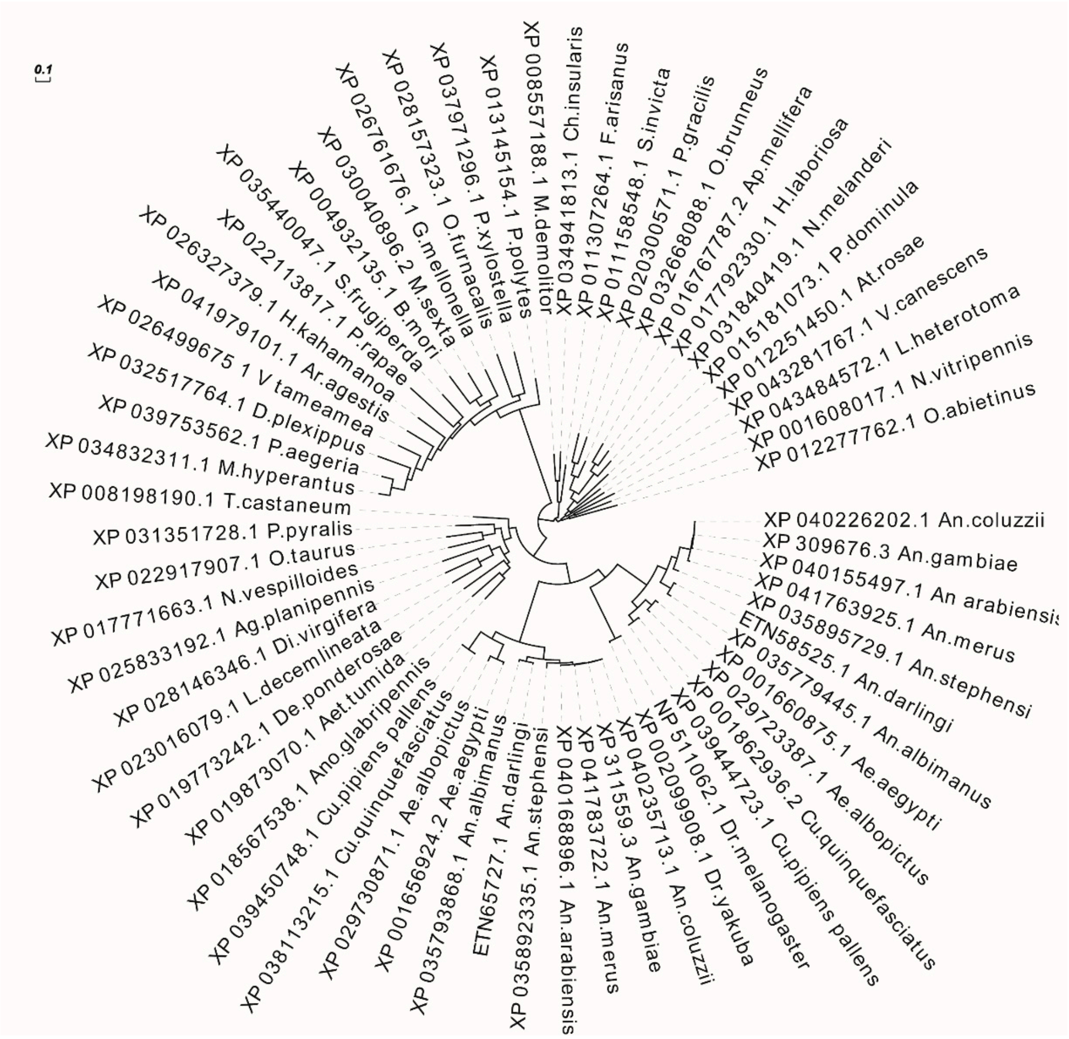

Figure S3. Detail of evolutionary relationships among amino acid sequences of AGT in Insecta. The evolutionary history was inferred by Bayesian analysis. The tree was rooted by Hymenoptera AGT as outgroup.

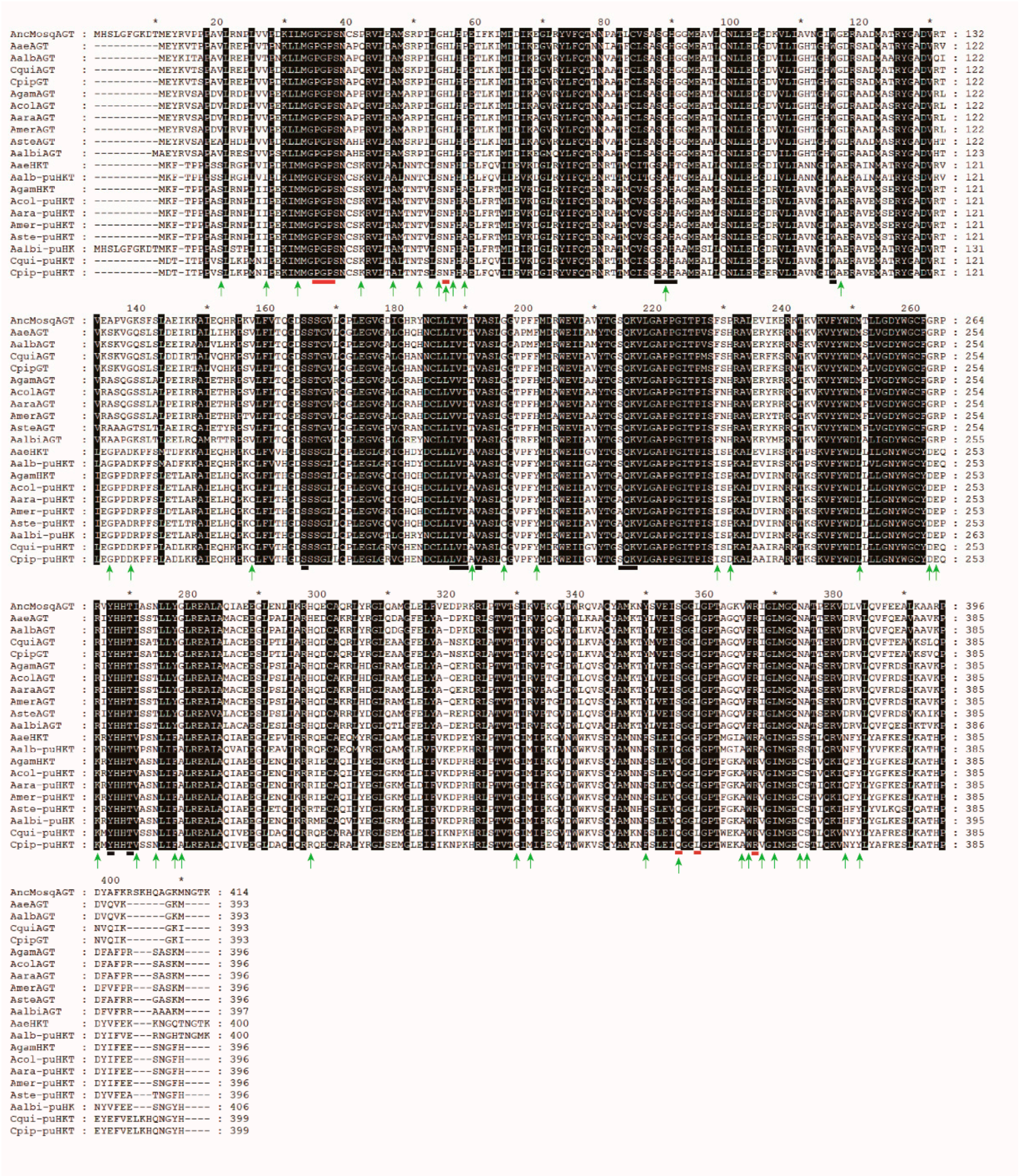

Figure S4. Multi sequence alignment of ancestral mosquito AGT and modern AGT. The black line markers below the residues represent the PLP binding site, and the red line markers represent the ligand binding site. Green arrows point to residues subjected to positive selection in HKT.

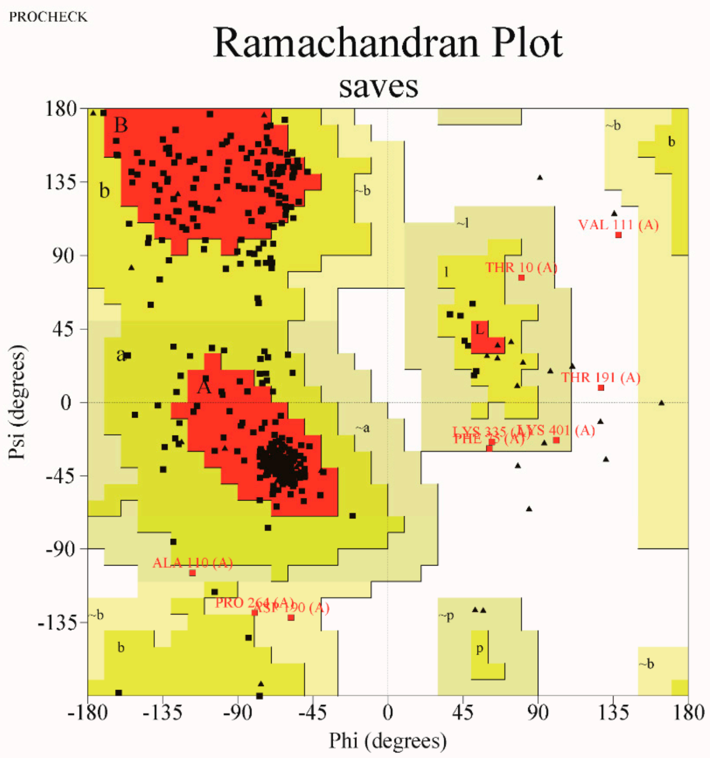

Plot statistics

Residues in most favoured regions [A,B,L]	273	78.7%
Residues in additional allowed regions [a,b,l,p]	66	19.0%
Residues in generously allowed regions [~a,~b,~l,~p]	6	1.7%
Residues in disallowed regions	2	0.6%
	----	------
Number of non-glycine and non-proline residues	347	100.0%
Number of end-residues (excl. Gly and Pro)	2	
Number of glycine residues (shown as triangles)	39	
Number of proline residues	26	

Total number of residues	414	

Based on an analysis of 118 structures of resolution of at least 2.0 Angstroms and R-factor no greater than 20%, a good quality model would be expected to have over 90% in the most favoured regions.

Figure S5. Ramachandran plot of newly built AncMosqAGT model.

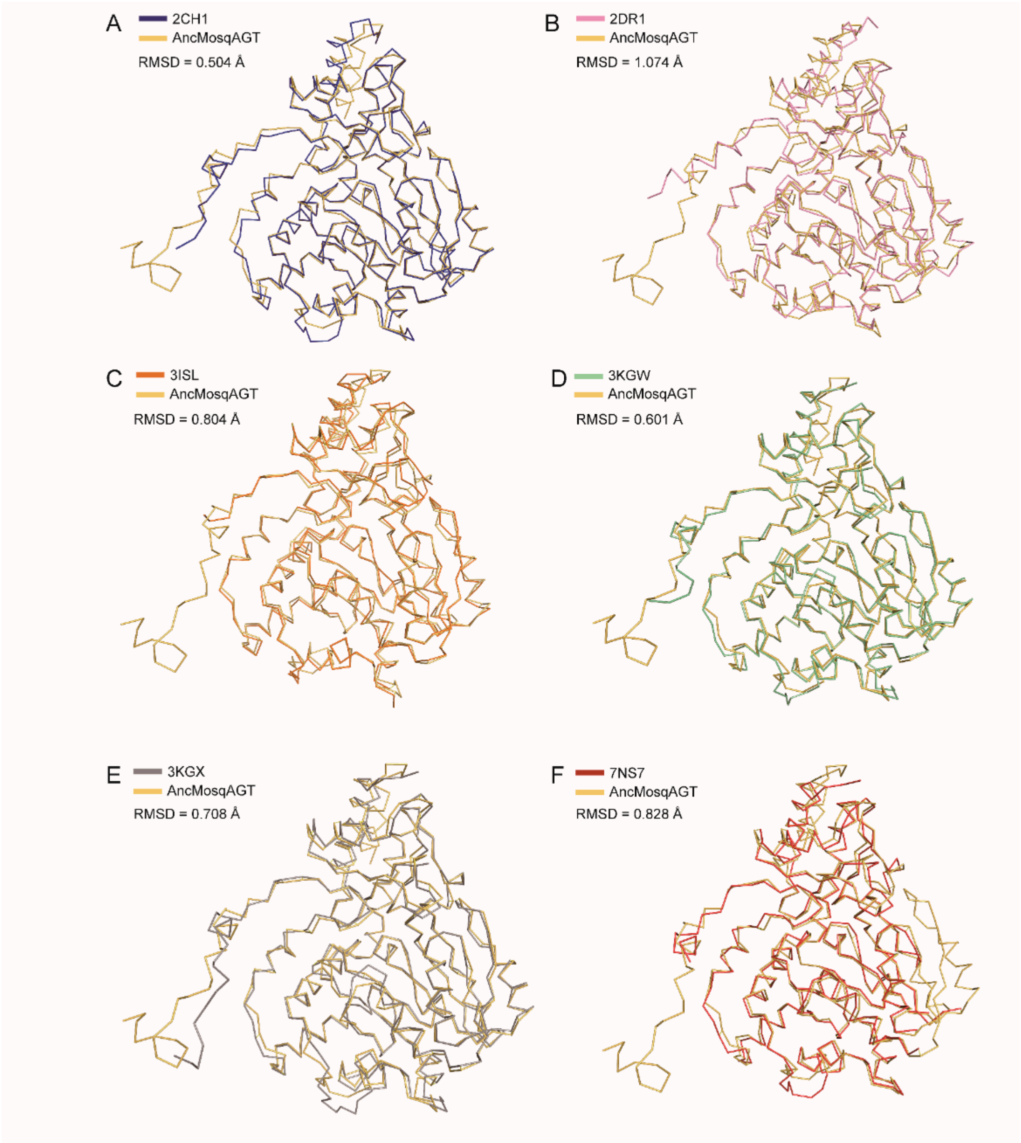

Figure S6. Superposition of newly built AncMosqAGT model and templates. RMSD value represents the root mean square deviation of the Cα atoms between model and templates (**A**–**F**).
